# Knowledge transfer in Tehran University of Medical Sciences: an academic example of a developing country

**DOI:** 10.1186/1748-5908-3-39

**Published:** 2008-08-26

**Authors:** Saharnaz Nedjat, Reza Majdzadeh, Jaleh Gholami, Sima Nedjat, Katayoun Maleki, Mostafa Qorbani, Mostafa Shokoohi, Mahnaz Ashoorkhani

**Affiliations:** 1 School of Public Health, Centre for Academic and Health Policy Research (CAHP), TUMS-KTE Study Group, Tehran University of Medical Sciences, Tehran, Iran; 2 Centre for Academic and Health Policy Research (CAHP), TUMS-KTE Study Group, Tehran University of Medical Sciences, Tehran, Iran; 3 School of Medicine, Golestan University of Medical Sciences, Golestan, Iran; 4 Graduate of School of Public Health, Tehran University of Medical Sciences, Tehran, Iran

## Abstract

**Background:**

In the past two decades, scientific publications in Iran have considerably increased their medical science content, and the number of articles published in ISI journals has doubled between 1997 and 2001. The aim of the present study was to determine how frequently knowledge transfer strategies were applied in Tehran University of Medical Sciences (TUMS). We were also interested in studying the determining factors leading to the type of strategy selected.

**Methodology:**

All TUMS research projects that had received grants from inside and outside the university in 2004, and were completed by the end of 2006, were included in the study. In total, 301 projects were examined, and data on each of the projects were collected by the research team using a standardized questionnaire. The projects' principle investigators filled out a second questionnaire. In all, 208 questionnaires were collected.

**Results:**

Researchers stated being more engaged in the passive strategies of knowledge transfer, especially those publishing in peer-reviewed journals. The mean score for the researchers' performance in passive and active strategies were 22% and 9% of the total score, respectively. Linear regression analysis showed that the passive strategy score decreased with the increase in the number of years working as a professional (p = 0.01) and personal interest as the only reason for choosing the research topic (p = 0.01). Regarding the active strategies of knowledge transfer, health system research studies significantly raised the score (p = 0.02) and 'executive responsibility' significantly lowered it (p = 0.03).

**Conclusion:**

As a study carried out in a Middle Eastern developing country, we see that, like many other universities in the world, many academicians still do not give priority to active strategies of knowledge transfer. Therefore, if 'linking knowledge to action' is necessary, it may also be necessary to introduce considerable changes in academic procedures and encouragement policies (*e.g.*, employment and promotion criteria of academic members).

## Background

'What happens to research-based findings after they are completed and published?' This is a question heard more often with the qualitative and quantitative development of research. In the 2004 World Health Organization report on 'knowledge for better health', 'linking research to action' was emphasized, and countries were asked to take serious steps in transferring research-based knowledge [1]. Knowledge transfer methods have been classified into active and passive strategies from researchers' perspective [2]. In passive strategies, the aim is diffusion and basically changing the awareness of the target audience. Normally, these activities are of importance in the academic environment, and are indicated by the publication of articles in peer-reviewed journals. Conversely, active strategies are based on interaction with the users of research results, and the possibility of behavior change is higher in these cases [3].

Iran's health systems infrastructure is what makes its medical research unique among other countries. In 1985, Iranian medical schools were integrated into the Ministry of Health, and the Ministry of Health and Medical Education (MOHME) was created. Under this infrastructure, education, research, and service delivery were unified [4], and it was expected that knowledge transfer would take place more effectively. In addition, in the past two decades the number of scientific publications in Iran has considerably increased [5], and the number of articles published in ISI journals with medical science content has doubled from 1997 to 2001 [6]. Tehran University of Medical Sciences (TUMS) has 1,250 academic members, or 12% of the country's medical academic members. Also, TUMS-affiliated researchers publish more than 30% of Iran's medical scientific articles in international databases.

The first objective of this study was to determine the frequency of various knowledge transfer activities applied by researchers at TUMS, and the second objective was to find the determining factors leading to the type of strategy ('active' or 'passive'). The findings of this study build a foundation upon which interventions in knowledge utilization can be studied in the future.

## Methods

### Data-gathering tools

The tools for data-gathering consisted of two sections: the data-gathering form (checklist), which was filled by the research team using research proposals and final reports [see Additional File 1], and the researcher's questionnaire (self-administered) which was sent to the principle investigators (a maximum of three times at one month intervals) [see Additional File 2].

The content validity of the questionnaire was approved after literature review and peer review. Pre-testing was done to assess feasibility; face validity, and reliability. A pilot study was performed on 10 data-gathering forms by studying 10 files and creating necessary changes. Also, 20 researchers completed the questionnaire twice at two week intervals to assess repeatability and internal consistency of the questions. The intra-class correlation indicator, which was considered the repeatability indicator, was 0.69 and 0.72 for the domains under study (active and passive strategies domains). The internal consistency (Cronbach's alpha) of these domains was 0.63 and 0.76. The questionnaire included the following variables: the percentage of time the participants allocated to research activities, the 'reasons for choosing the research topic', and the researchers' performances in knowledge transfer activities.

In order to study their role in knowledge transfer activities, researchers were asked to mark all the activities they had carried out in the field of knowledge transfer (including active and passive strategies) from a list that was presented to them. We also left an open-ended question for the activities that were not listed in the above-mentioned questions. A score of zero was given if the activity was not carried out; a score of one if it was performed once, and a score of two if it was done more than once. The total score then was summed for each research activity. The following activities were considered 'passive' strategies of knowledge transfer: delivery of the project report or its summary to users; preparing articles and publishing reports in domestic and international peer-reviewed journals; displaying results on a website; posting or e-mailing articles or reports and/or their summaries for stakeholders without their request; and presenting the results in domestic or international conferences and seminars, and/or publishing research results in newspapers. The activities that were considered 'active' for knowledge transfer were as follows: preparation and delivery of content in plain language; holding briefings with stakeholders for presentation of research results; and presenting results to the media and participation in interviews. Also, we asked researchers to note the percentage of time, or 'percent effort' they allocated to each activity, including research, education, clinical service delivery, executive responsibilities, and others. Researchers were then asked to estimate their percent effort in a way that the sum would be equal to 100 (Question 6, Additional File 2).

### Population under study

All TUMS research projects that received grants from inside and outside the university in 2004 and were completed by the time this study was performed (the second half of 2006) were studied. The number of research projects that met the inclusion criteria of this study was 315, out of which the data-gathering forms were completed for 301 projects (95.6%). Fourteen projects were not entered into the study due to unavailability of files. The researcher questionnaire was then sent to the principle investigators of these projects, and 208 questionnaires were collected. Non-responders included 32 researchers who were unavailable and 75 who did not respond after three requests, giving a final response rate of 74%. In order to assess whether a significant difference existed between those researchers who responded to the questionnaire and those who did not, their project proposal forms were compared. This was carried out by reviewing the 'problem statement' of the research proposals. We observed that 24% of the individuals who did not respond to the questionnaire mentioned choosing their topics on the basis of needs assessment. This proportion was 17% for those who responded to the questionnaire. The difference between these two groups was not statistically significant (p = 0.17).

### Data analysis

Apart from the usual descriptive statistics for data analysis, multi-variable linear regression was used to control the effect of the potential confounders, including gender, number of years working as a professional, and tenure status (half-time or full-time). For these purposes, the data were analyzed with SPSS/version 11.5 statistical software.

### Ethical considerations

This study was approved by the TUMS ethics review board as part of the reviewing process of TUMS research projects.

## Results

### Population under study

A total of 208 researchers participated, 130 of whom were male (62.5%). The age range was 25 to 72 years, and the mean age was 45.6 years (SD = 9.4). Regarding academic rank, 15% of researchers were non-academic members, 7% were instructors, and 33%, 26%, and 19% were assistant, associate, or full professors, respectively. Employment status included 181 (87%) full-time employees and 10 (4.8%) part-time employees. The remaining respondents did not answer this question. Number of years working as a professional ranged from one to 43 years, and the mean number of years working in the university was 14.3 (SD = 8.5). Aside from education and research, 123 individuals had executive responsibilities such as management of a hospital, school, department or ward, research deputy of the school, and/or research center, *etc*. Seventy-two individuals (34.6%) were involved solely in education and/or research.

The research projects were divided into three groups according to proposal type. There are two formats of proposals at TUMS. One is health system research, in which the end-users are policy makers, managers, and health system experts. The other format is for clinical and basic studies, where the researcher chooses which category the proposal most addresses. Nevertheless we confirmed the validity of their choice by checking whether the targets of research were clinical practitioners, basic researchers, or health system researchers. (*e.g.*, a study that is carried out to better understand a topic and has no immediate clinical application is a basic study, a study whose results are directly used by the clinician is a clinical study, and a study whose results are used by managers and policy makers is a health system research study). The researchers were then divided into basic sciences (46 cases), clinical studies (101 cases), and health system research (61 cases). Comparing the duration of time allocated to research in these three groups showed that the mean percentage of time allocated to research in the basic sciences group was 41% (SD = 22), and a significant difference (p < 0.001) was observed between this group and the clinical (27%, SD = 16) and health system research (30%, SD = 19)groups, respectively. Researchers were asked about their reasons for choosing the research topic. Thirty-one participants (14.9%) stated 'personal interest or repeating others research'. This proportion was 23.9% for the basic sciences, 7.9% for clinical studies, and 19.7% in health system researchers (p = 0.02), whereas the remainder mentioned choosing their topics based on 'other organizations request or needs assessment'.

### The knowledge transfer status (First objective)

#### Information gathered from the self-administered questionnaire

Table 1 shows researcher behavior with respect to passive strategies of knowledge transfer. The first four rows of this table (publishing articles in peer-reviewed journals and presentations at conferences) are criteria which are valued in the assessment of academic staff members, whereas the other criteria are of no value. In all types of research, the researchers stated that publishing in peer-reviewed journals had the greatest impact in disseminating research results. Most basic science research was sent to international journals (71.7%), and most clinical and health system research was sent to domestic journals (74.3% and 57.3% respectively). The last row of this table shows that the least effort made by researchers is for publishing research results in newspapers, which was found in only eight out of 208 cases (4%).

**Table 1:** 'Passive' knowledge transfer strategies of TUMS researchers, based on the type of research.

Strategy	Basic		Clinical		Health system		Total	
	
	Number n = 46	Percent 22.1	Number n = 101	Percent 48.6	Number n = 61	Percent 29.3	Number n = 208	Percent 100
Publicaation of articles in domestic journals	20	43.5	75	74.3	35	57.4	130	62.5
Publication of articles in international journals	33	71.7	55	54.5	13	21.3	101	48.6
Presenting research results in conferences, seminars, and domestic meetings	20	43.5	55	41.0	25	41.0	100	48.1
Presenting research results in conferences, seminars, and international meetings	22	47.8	39	38.6	10	16.4	71	34.1
Sending the complete report of the research project to users	21	45.7	40	39.6	32	52.5	93	44.7
Sending a summary report of the project to users	19	41.3	45	44.6	29	47.5	93	44.7
Displaying the results on the web site	13	28.3	11	10.9	15	24.6	39	18.8
Mailing or emailing articles, reports, or summaries for stakeholders without their request	4	8.7	4	4.0	7	11.5	15	7.2
Publishing research results in newspapers (in which the general public is interested)	1	2.2	4	4.0	3	4.9	8	3.8

Table 2 shows the active strategies of knowledge transfer. In all three fields of basic, clinical, and health system research, the step taken most often was 'preparing and delivering text in plain language'. 'Holding briefings with stakeholders for presentation of research results was also frequently cited for health system research, but presenting results in the media was of little significance.

**Table 2:** 'Active' knowledge transfer strategies of TUMS researchers, based on the type of research.

Strategy	Basic		Clinical		Health		Total	
	
	Number n = 46	Percent 22.1	Number n = 101	Percent 48.6	Number n = 61	Percent 29.3	Number n = 208	Percent 100
Preparation and delivery of texts suitable to the users (such as plain writings for patients, special texts for managers, practical reports for clinical and lab colleagues, special reports for industrial managers or academics)	7	15.2	11	10.9	14	23.0	32	15.4
Presenting results to reporters, radio and TV for dissemination in the media and participation in interviews	2	4.3	8	7.9	6	9.8	16	7.7
Holding briefings with stakeholders for presentation of research results	2	4.3	6	5.9	13	21.3	21	10.1

#### Information gathered from files (research proposals and final reports)

A review of 301 research proposals showed that the total budget of the projects under study was a little less than US$1,290,000: US$324,280 for health system research, US$488,030 for clinical research and US$471,380 for basic research. The total expense considered for knowledge transfer for 301 projects was approximately US$13,200: US$12,790 for health system research, US$376 for clinical research, and none for basic research. This amount was spent on only seven cases (2.3%), of which five were health system research and two were clinical research. In this analysis, a significant difference was found to exist between the groups in this regard, and in the *post hoc *analysis this difference was insignificant among the clinical and basic research groups alone, but the cost for knowledge transfer activities in health system research was significantly higher than that for clinical and basic sciences.

A review of the project final reports showed that in 142 final reports and/or project summaries (47.2%) the target audience had been identified. In this case, a significant difference did not exist between the three groups (basic, clinical, and health system research) (p = 0.28). In 150 project reports (49.8%), a clear suggestion had been made to the target audience. Even here a significant difference did not exist between the groups (p = 0.11). Of all 150 final reports examined, 87.3% of these suggestions had somehow pointed to the manner of the measure to be taken, but in 37.3% it had been made clear as to who had to take what measure.

#### Determinant factors of knowledge transfer (Second objective)

In the 'passive' strategies section, the maximum score attainable was 18. The mean score for researchers' performance was 4.00 (SD = 3.03) that formed 22% of the total score. The maximum score attainable in the active strategies was six and the mean score of the researchers' performance in these strategies was 0.54 (SD = 1.02), which consisted of only 9% of the total score. Table 3 and 4 show the results of a linear regression analysis with the 'Enter' method. As shown in tables 3 and 4, the dependent variables in these regressions are the scores of passive and active strategies, respectively. These scores were obtained from the number of activities the researchers claimed to have carried out, whereas the independent variables included gender, number of years working as a professional, tenure status (half-time or full-time), reasons for choosing the research topic, and type of research (basic sciences were taken as reference with respect to clinical and health system research). Controlling the confounding variables, regression coefficients show the effect of each of these variables on passive and active strategy scores. In table 3, the number of years working as a professional and health system research (as compared to basic research) have a significant inverse relationship with the passive strategy scores, whereas choice of the research topic based on other organizations' request or needs assessment increases the score significantly. According to the results of the linear regression analysis in table 4, health system research and executive responsibilities had a significant effect on this score.

**Table 3:** The relation of independent variables on the score obtained on 'passive' strategies of knowledge transfer in the linear regression analysis.

	Regression coefficient*	Standard error	P-value
Sex (male/female)	0.00	0.46	0.99
Associate professor (in comparison to an assistant professor)	-0.28	0.57	0.62
Professor (in comparison to an assistant professor)	0.71	0.68	0.30
Instructor (in comparison to an assistant professor)	-1.09	0.91	0.23
Non-academic member (in comparison to an assistant professor)	0.61	0.91	0.50
Tenure status (full time/half time)	-1.02	1.18	0.39
Number of years working as a professional	-0.08	0.03	0.01
Executive responsibility (has/hasn't)	-0.65	0.47	0.17
Time allocated to research (percentage of total time)	0.01	0.01	0.39
Reasons for choosing the research topic (choice based on other organizations' request or need assessment vs. personal interest or repeating others research)	1.68	0.63	0.01
Clinical researches (in comparison to basic science researches)	-0.74	0.65	0.39
Health researches (in comparison to basic science researches)	-1.55	0.68	0.02

*These coefficients represent the change in the total score of passive strategies, where the maximum score attainable is 18.

**Table 4:** The relation of independent variables on the score obtained on 'active' strategies of knowledge transfer in the linear regression analysis.

	Regression coefficient*	Standard error	P-value
Sex (male/female)	-0.09	0.16	0.59
Associate professor (in comparison to an assistant professor)	0.09	0.20	0.67
Professor (in comparison to an assistant professor)	0.31	0.24	0.18
Instructor (in comparison to an assistant professor)	0.13	0.31	0.68
Non-academic member (in comparison to an assistant professor)	0.12	0.31	0.70
Tenure status (full time/half time)	-0.18	0.41	0.66
Number of years working as a professional	-0.02	0.01	0.08
Executive responsibility (has/hasn't)	-0.36	0.16	0.03
Time allocated to research (percentage of total time)	0.01	0.01	0.33
Reasons for choosing the research topic (choice based on other organizations' request or need assessment vs. personal interest or repeating others research)	0.19	0.22	0.39
Clinical researches (in comparison to basic science researches)	-0.04	0.22	0.87
Health researches (in comparison to basic science researches)	0.51	0.23	0.02

*These coefficients represent the change in the total score of active strategies, where the maximum score attainable is 6.

## Discussion

This study shows that passive strategies hold a greater share of knowledge transfer activities as compared to active ones in TUMS. While TUMS researchers have gained 22% of the total score for passive strategies of knowledge transfer (including preparation of articles for publication in domestic and international peer-reviewed journals, presenting research results at conferences and seminars, *etc*), when it comes to active strategies of knowledge transfer (preparation and delivery of texts suitable to the users, presenting results to mass media, and holding briefings with stakeholders) this percentage amounts to 9% of the total score. The result is that the score obtained for passive strategies of knowledge transfer is 2.44 times greater than the scores obtained for active strategies.

Regarding publication of results in journals, according to the research regulations of TUMS at the time of this study, sending at least one article for publication from each project was one of the requirements for completing the project. This is why publication of articles in peer-reviewed journals is the most common knowledge transfer activity. According to table 1, basic science research studies are published more in international journals than in domestic journals as compared to health system research. This may be because basic science research is less dependent on the location of research. On the other hand, health system research studies that are more dependent on cultural, social, economic, and other contextual factors target domestic journals more than international ones.

When examining other passive strategies of knowledge transfer we observed that less than 19% of the researchers have displayed the results of their research on websites. The other point worth mentioning is that less than 4% of research results were published in newspapers. Newspapers and websites are important because they have broad geographical coverage and transcend time barriers, even though the evidence should be considered before presenting it to the media; not every research result can be disseminated. Tables 1 and 2 show the performance of TUMS' researchers is in accordance with the requirements of the academic promotion criteria. This emphasizes that incentive policies (recruitment, academic members' promotion, and granting financial rewards for publishing articles) are effective. On the contrary, other matters that can lead to implementation of research findings have not received similar attention. In fact, the current state of knowledge production dominant in this university (like most universities in the world) is passive, and for strengthening the connection of 'linking knowledge to action', basic changes are needed.

Valuing scientific productions (such as publishing articles in peer-reviewed journals and presentation of material at scientific conferences) are among the known factors affecting the knowledge transfer activities of academics [7-12]. The known methods of valuing are employment and promotion [8,13,14]. When matters such as professional progress are solely dependent on publishing in specialized frameworks, people are not motivated enough in transferring knowledge, and guaranteeing its utilization. For the sake of meeting communities' needs, current efforts are being made to revise the promotion and employment criteria from a new perspective [15-17]. On the other hand, intrinsic motivations such as researchers' perceptions, values, and beliefs are influential in this field; how these beliefs are shaped and to what extent they are influenced by education are matters which demand deeper qualitative approaches [18].

Regarding tables 3 and 4, we note that the method of summing up the scores of knowledge transfer activities as equal weight for various cases is a simple and optional approach. Linear regression analysis was done by entering all variables into the model. This type of analysis was chosen because, compared to other models that try to keep fewer variables in the final model, it has an exploratory aspect, and from the authors' point of view a better understanding of the variables in this field is necessary.

However, the result of the linear regression analysis showed that the scores of passive strategies of knowledge transfer decreased with the number of years working as a professional. That is, considering that the other variables are constant, with every one-year increase in number of years working as a professional, this score decreases by 0.08. The relationship between choosing the research topic (choice based on other organizations' request or need assessment versus personal interest or repeating others research) and the passive strategy score is positive. The passive strategy scores increase by 1.68 as a result of change of 'reasons for choosing the research topic' from 'personal interest or repeating others researches' to 'choice based on other organizations' request or need assessment'. The health system researchers also registered a lower score as compared to the basic science researchers, which leads to a 1.55 reduction in the passive strategy score.

Where active strategies are concerned, two variables were significant: First, executive responsibilities can significantly reduce the active strategies score by 0.36. This can be explained by the shortage of time this group is faced with. Second, as compared to basic science research, health system research increased the active strategy score by 0.51.

As shown in the tables, health system research registered lower scores in the passive strategies of knowledge transfer as compared to basic sciences, whereas in the active field of strategies the reverse was true. The scores registered by health system research were higher than basic sciences.

Studies of researchers from other countries have shown differences in knowledge transfer activities among various specialties. In a study done on researchers in Canada it was seen that applied science researchers use plain and engaged dissemination measures more than basic science researchers. Apart from the field of research (applied or basic) the researchers' working locations (medical school and others) have also been taken into consideration. Comparing the various methods of knowledge transfer, both these variables were shown to be significantly effective. Their interaction has also shown to be effective in the number of publications in this study [19].

After studying the final project reports, it was shown that almost 50% of them had proposed a suggestion for utilization of results (although a formal compulsory framework does not exist for writing the final report and having an actionable message). This shows that researchers need to pay more attention to knowledge transfer and that by valuing activities in this field, results can be properly utilized. Also, the target audiences of these messages were clear in 47.2% of cases, even though there is no compulsion for mentioning the target audiences. This shows that if researchers receive basic training for increasing their communication skills we will achieve more satisfactory results. This matter has been mentioned in other references and also been advised [20].

Review of the research proposals showed that in only 2.3% of the 301 cases under study, expenses for knowledge transfer activities had been considered, amounting to 1% of the funds requested. There are two reasons for this observation: Some researchers fail to consider knowledge transfer to be a part of research at all, and those who evaluate the cost of research (proposal reviewers at TUMS) find these costs unacceptable.

No doubt knowledge transfer activities require financial resources, be it in the form of cash paid for direct costs (such as preparation and handing out pamphlets or the cost of setting up meetings), or indirect costs (such as purchasing knowledge transfer services). Many authors have stated the lack of these facilities and funds to be potential barriers to the knowledge transfer process [8,11,13,21,22].

Because many of the study's data are based on the self-administered questionnaire, it is possible that responders may have overestimated their knowledge transfer activities. This may be due to the social undesirability of the answers that point to lack of knowledge transfer activity. Therefore this study may be prone to information bias in describing knowledge transfer activities, despite the fact that the questionnaire had been evaluated for repeatability and internal consistency prior to the study. This information bias can affect the first descriptive objective but we do not assume the second objective, *i.e.*, study of determinant factors, to be biased as a result of this.

## Conclusion

This study was carried out in one of the universities of a Middle Eastern developing country. Here we observe that, like many other universities in the world, many academicians still do not give priority to active strategies. Even though previous studies have shown that many factors affect the facilitation of knowledge transfer in the university [23], but the matter of giving priority to knowledge transfer largely depends on academic priorities which are shown in its policies. Therefore if knowledge transfer is to be a priority, it is necessary to introduce considerable changes in academic procedures and incentive policies (*e.g.*, employment qualifications and promotion criteria). The universities also need to show commitment to knowledge transfer. This means that apart from creating the necessary motivation in researchers, support mechanisms should also be provided.

As previously mentioned, the main feature of Iran's medical research is that research and service delivery are under a common stewardship, which is an aftermath of integration of medical universities into the ministry of health. Therefore, it will be interesting to study the impact of integration on knowledge transfer in the future.

## Competing interests

The authors declare that they have no competing interests.

## Authors' contributions

SN and RM participated in the design, statistical analysis, and manuscript writing. JG designed and conducted the study. SN, MG, MS, and MA gathered the data. KM assisted in interpreting the statistical analysis and manuscript writing. All authors approved the final manuscript.

## Supplementary Material

Additional file 1 The Research Questionnaire (checklist) Click here for file

Additional file 2 Researcher's Questionnaire Click here for file
