# Supplementary material for: Knowledge transfer in Tehran University of Medical Sciences: an academic example of a developing country
Source: Implement Sci. 2008 Aug 26;3:39. doi: 10.1186/1748-5908-3-39 (PMC2538542; doi:10.1186/1748-5908-3-39)
Supplement: Additional file 1 — The Research Questionnaire (checklist) [file 1748-5908-3-39-S1.doc]

**Additional file 1**

**Research Questionnaire (checklist)**

The code of the article

1) The article title:

|  |
| --- |

2)

| Name of the journal:  Publication year: |
| --- |

3) Authors' names:

|  | Name and Surname | Scientific level | Field of study | Location of service |
| --- | --- | --- | --- | --- |
| Corresponding author |  |  |  |  |
| Rest of the authors, consecutively |  |  |  |  |
|  |  |  |  |
|  |  |  |  |
|  |  |  |  |
|  |  |  |  |
|  |  |  |  |
|  |  |  |  |

| **Clinical Study:** A study whose results are directly used by the clinician  **Health System Research Study:** A study whose results are used by managers and policy makers  **Basic Study:** A study that is done for the understanding of a topic and has no immediate application |
| --- |

4) In which field has the study been done?

1- Clinical **❑** 2- Health System Research (HSR) **❑**

3- Basic science research **❑**

- **Abstract:**

5) Has a clear practical suggestion been made to the target audience in the abstract of the article?

1- Yes **❑** 2- No **❑**

6) Have the target audiences been directly addressed in the abstract of the article?

1- Yes **❑** 2- No **❑**

- **Introduction:**

7) Has the utilization of the results of this project in the health system (clinical or managerial) been directly mentioned in the introduction?

1- Yes **❑** 2- No **❑**

| By target audiences we mean: the people, special groups of the society, patients, health managers and policy makers, managers and policy makers of other organizations, other researchers, service providers (clinical, laboratory, health *etc*). |
| --- |

8) Have the target audiences of the project been directly addressed in the introduction?

1- Yes **❑** 2- No **❑**

- **Methodology:**

9) Type of study:

1- Case series **❑** 2- Cross sectional **❑**  3- Case control **❑** 4- Cohort**❑**

5- Interventional and/or Clinical trial **❑** 6- Experimental**❑** 7- Studies for manufacturing drugs or medical equipment **❑** 8- Inaugurating a scientific implementing system **❑**

9- Test examinations **❑** 10- Method examinations **❑**

11- Qualitative studies **❑** 12- Health system managerial studies **❑**

13- Software design **❑** 14- Systematic review **❑** 15- Review article **❑** 16- Other **❑** (Please explain)

| Example of an actionable message: Unemployment is directly related to poor health, and this relationship is independent of other factors such as lifestyle or inactivity. |
| --- |

- **Discussion and Conclusion:**

10) Has an "actionable message" been mentioned in the discussion and conclusion of the study?

1- Yes **❑** 2- No **❑**

| Context of the message: |
| --- |

11) Have the target audiences been clearly addressed in the discussion and conclusion portion?

1- Yes **❑** 2- No **❑**

- **Acknowledgements:**

12) Has collaboration or financial support of executive organizations such as the Ministry of Health or relative organizations been mentioned in the Acknowledgements?

1- Yes **❑** 2- No **❑** 3-Doesn’t have acknowledgements **❑**
